# Development of Class IIa Bacteriocins as Therapeutic Agents

**DOI:** 10.1155/2012/386410

**Published:** 2011-11-30

**Authors:** Christopher T. Lohans, John C. Vederas

**Affiliations:** Department of Chemistry, University of Alberta, Edmonton, AB, Canada T6G 2G2

## Abstract

Class IIa bacteriocins have been primarily explored as natural food preservatives, but there is much interest in exploring the application of these peptides as therapeutic antimicrobial agents. Bacteriocins of this class possess antimicrobial activity against several important human pathogens. Therefore, the therapeutic development of these bacteriocins will be reviewed. Biological and chemical modifications to both stabilize and increase the potency of bacteriocins are discussed, as well as the optimization of their production and purification. The suitability of bacteriocins as pharmaceuticals is explored through determinations of cytotoxicity, effects on the natural microbiota, and *in vivo* efficacy in mouse models. Recent results suggest that class IIa bacteriocins show promise as a class of therapeutic agents.

## 1. Introduction

Bacteriocins are natural peptides secreted by many varieties of bacteria for the purpose of killing other bacteria. This provides them with a competitive advantage in their environment, eliminating competitors to gain resources. These peptides are ribosomally synthesized, although some are extensively posttranslationally modified. 

The classification system for bacteriocins has been subject to ongoing revision [1–3]. However, bacteriocins from Gram-positive bacteria are generally classified according to size, structure, and modifications. Class I bacteriocins are the lantibiotics, which are highly posttranslationally modified peptides containing lanthionine and methyllanthionine residues. Class II consists of small peptides that do not contain modified residues. Cotter et al. suggested to divide class II bacteriocins into several subclasses: class IIa (pediocin-like bacteriocins), class IIb (two-peptide bacteriocins), and class IIc (circular bacteriocins) [3]. However, others have suggested to consider circular bacteriocins as a separate class [4]. Nonbacteriocin lytic proteins, termed bacteriolysins (also referred to as class III bacteriocins), are large and heat-labile proteins with a distinct mechanism of action from other Gram-positive bacteriocins [3]. 

**Figure 1:**
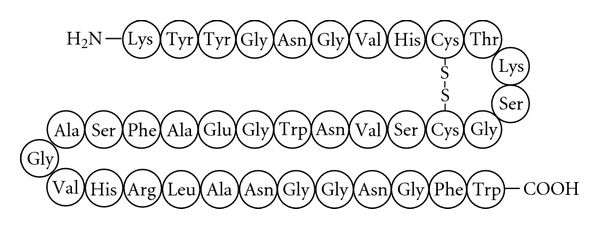
A representation of class IIa bacteriocin leucocin A, with the YGNGV consensus sequence and an N-terminal disulfide bridge.

Class IIa bacteriocins are generally from 37 to 48 amino acids long, and are characterized by several features. Although they do not have broad spectrum antimicrobial activity compared to other antibiotics, they are particularly potent inhibitors of *Listeria* species, showing activity at low nanomolar concentrations [5]. They are heat-stable, and not posttranslationally modified beyond the proteolytic removal of a leader peptide and the formation of a conserved N-terminal disulfide bridge (although some members contain an additional C-terminal disulfide bridge). The N-terminal region contains a characteristic YGNGV amino acid sequence, although variants with the alternate YGNGL sequence have been classified in class IIa [6]. A representative class IIa bacteriocin is shown in Figure 1. There have been a number of thorough reviews describing aspects of the genetics, biosynthesis, immunity, structure, mode of action, and the application of class IIa bacteriocins to foods [7–13].

Briefly, class IIa bacteriocins kill susceptible bacteria by forming pores in their membranes, resulting in the loss of the proton-motive force and depletion of ATP [14]. It is thought that these cationic bacteriocins are drawn to bacterial cells through an initial electrostatic interaction [15]. Then, the amphiphilic C-terminal *α*-helix inserts into the membrane, wherein the bacteriocin induces the formation of hydrophilic pores. This mechanism of action is reliant on a mannose phosphotransferase (MPT) protein complex found in the membranes of susceptible organisms, but the exact nature of this interaction is not yet clear [16–18]. This is covered in more detail by Drider et al. [12] and Nissen-Meyer et al. [19].

**Figure 2:**
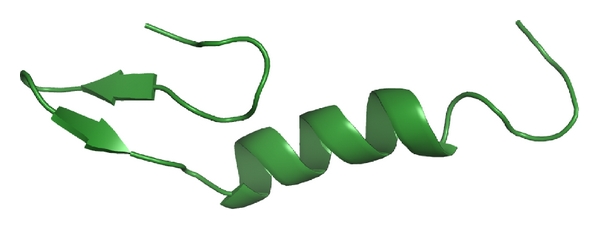
The NMR solution structure of leucocin A [20].

Structurally, the N-termini of class IIa bacteriocins tend to exhibit a three-strand antiparallel beta-sheet structure rigidified by a disulfide bridge. The C-terminal region shows an amphiphilic helix terminating in a hairpin structure. In aqueous conditions, class IIa bacteriocins are randomly structured. However, membrane-mimicking conditions such as dodecylphosphocholine micelles or trifluoroethanol induce structure formation [20]. This is not unexpected as their mode of action involves membrane permeabilization [14]. The NMR solution structures of class IIa bacteriocins leucocin A (shown in Figure 2) [20], carnobacteriocin B2 [21] and its precursor precarnobacteriocin B2 [22], sakacin P [23], and curvacin P [24] have been solved to date. 

Much of the research on class IIa bacteriocins has focused on their application for food preservation. While they may be well-suited for this purpose, there is a growing body of research exploring the prospect of using these bacteriocins as *in vivo* therapeutic agents. Bacteriocins are a promising substitute for conventional antibiotics for several reasons. The restricted target specificity of some bacteriocins minimizes their impact on commensal microbiota and may decrease the threat of opportunistic pathogens. Furthermore, most bacteriocins are active at low concentrations, and their degradation products are easily metabolized by the body. With the development of resistance to many important antibiotics, another tool for fighting bacteria is invaluable. 

Class IIa bacteriocins are active against several important human pathogens. Perhaps most promising is their activity against the foodborne pathogen *Listeria monocytogenes*, the deadliest bacterial source of food poisoning [25]. Up to 30% of foodborne infections by *L. monocytogenes* in high-risk individuals are fatal. Other bacterial foodborne pathogens inhibited by some class IIa bacteriocins include *Bacillus cereus, Clostridium botulinum*, and *C. perfringens* [5]. 

Beyond foodborne pathogens, class IIa bacteriocins are also active against other human pathogens, such as vancomycin-resistant enterococci [26] and the opportunistic pathogen *Staphylococcus aureus *[5]. Although bacteria sensitive to class IIa bacteriocins are almost exclusively Gram-positive, the Gram-negative opportunistic pathogen *Aeromonas hydrophila* is also inhibited [27]. Bacteriocins from this class also show other potentially therapeutic properties as antineoplastic [28, 29] and antiviral [30] agents.

The potential of other groups of bacteriocins such as lantibiotics, colicins, and microcins as oral and gastrointestinal antibiotics has been reviewed by Kirkup [31]. Focusing on bacteriocins from Gram-positive bacteria, there have been successes with the administration of either lantibiotic-producing bacteria [26] or purified lantibiotics [32–38] for the treatment of infections by several different pathogens. However, less is known about the *in vivo* use of class IIa bacteriocins. 

One method for the therapeutic use of bacteriocins is to introduce bacteriocinogenic bacteria to the gastrointestinal tract as probiotics, which has yielded positive results. Frequently, the mechanisms by which probiotic bacteria benefit the host are not well characterized, but convincing evidence has been put forth by Corr et al. for the production of class IIb bacteriocin Abp118 *in vivo* [39]. Generally, the introduction of bacteriocinogenic bacteria prior to infection with a pathogen has been more effective [26, 39] than the concomitant introduction of both species [40]. This suggests that probiotic strains may be valuable for prophylactic purposes, but less suited for treating preexisting infections. 

Indeed, introduction of a bacteriocin either concomitantly with the infectious agent or postchallenge has proven effective [32–38]. A variety of administration methods have been used successfully: subcutaneous, intravenous, intranasal, intragastric, intraperitoneal, and topical [41]. The efficacy of the different methods has not been directly compared and likely depends on the pathogen targeted. Furthermore, some of these methods may be unnecessarily invasive for use in humans, with oral administration being preferred. Although the possibility exists of using crude bacteriocin extract instead of purified bacteriocin, the introduction of complex mixtures into a human may be hazardous and less reproducible. Instead, this paper will focus on the administration of purified class IIa bacteriocin.

Compared to other classes of Gram-positive bacteriocins, the engineering of improved class IIa bacteriocins is somewhat simplified due to their unmodified nature. Creating analogues, by biological or chemical means, does not require implementation of the thioether bridges found in lantibiotics or the cyclization required for circular bacteriocins. Nor does the recombinant expression of class IIa bacteriocins require the biosynthetic machinery, such as dehydratases and cyclases, required for some other bacteriocins. This also allows for the production of class IIa bacteriocins as fusion proteins, a means of increasing production levels and simplifying purifications. 

This paper will explore different aspects of the development of class IIa bacteriocins as therapeutic agents for *in vivo *utilization. The first section discusses attempts to design bacteriocins and bacteriocin analogues with increased stability and potency. Next, methods for improving production and purification of large amounts of bacteriocin from fermentation and recombinant expression will be explored. Finally, the suitability of class IIa bacteriocins for therapeutic use, based on studies testing cytotoxicity, stability, the development of resistance, and the *in vivo *potential of class IIa bacteriocins will be examined.

## 2. Engineering Class IIa Bacteriocins for Increased Stability and Potency

The structure-function relationship of class IIa bacteriocins has been well studied, and its implications for their mode of action has been well reviewed [8, 12, 19]. This paper focuses on structure-function as it contributes to the development of improved therapeutics. Specifically, engineering bacteriocins to increase their stability, potency, and spectrum of activity, such that they are more suitable for *in vivo* utilization and other applications will be discussed.

The introduction of an additional disulfide bridge likely has the effect of rigidifying a specific conformation and could result in improved bacteriocin activity. There is a subgroup of class IIa bacteriocins, including pediocin PA-1, that contain an additional disulfide bridge near the C-terminus. The effect of introducing a C-terminal disulfide bridge into sakacin P, a bacteriocin containing only the conserved N-terminal disulfide bridge, was examined [42]. This modification broadened its spectrum of antimicrobial activity in addition to decreasing the detrimental effect of increased temperature on potency. The C-terminus has been otherwise associated with the target specificity of class IIa bacteriocins [43]. Notably, this sakacin P mutant was found to retain much of its activity at 37°C compared to the natural peptide, and thus is more effective at human physiological temperature [42].

The necessity of the N-terminal disulfide bridge for activity in class IIa bacteriocins has also been explored. Removal of this disulfide bridge could render bacteriocins more stable in reductive environments. Substitution of cysteines 9 and 14 of leucocin A [44] and mesentericin Y105 [45] with serines resulted in a complete loss of activity. However, replacement with hydrophobic residues such as allylglycine, norvaline, and phenylalanine resulted in retention of activity in leucocin A [46]. Furthermore, the replacement of the disulfide bridge with a carbocycle also yielded a biologically active peptide, although the activity was decreased by an order of magnitude [44]. However, the substitution of cysteines 9 and 14 of pediocin PA-1 with allylglycine and phenylalanine residues resulted in no observable activity [46]. This work has been discussed further in a mini-review by Sit and Vederas [47].

Class IIa bacteriocins may also be stabilized by simple amino acid substitutions. Methionine-31 of pediocin PA-1 was found to oxidize over time with an accompanying loss of activity [48]. Mutation of this residue to leucine, isoleucine or alanine resulted in only minor decreases in potency while stabilizing the mutant [48]. Similarly, a 4- to 8-fold decrease in activity was reported for carnobacteriocin BM1 due to an oxidized methionine residue [49], but replacement with a valine residue yielded a mutant with comparable activity [50]. However, in some cases substitution of only a single amino acid residue in class IIa bacteriocins results in dramatically decreased activity relative to their wild-type counterparts [51].

Consideration of the mode of action of class IIa bacteriocins may permit the rational design of mutants with increased potency. Enhancing the net positive charge of a bacteriocin may be expected to promote the initial electrostatic interaction with the membrane of the target and thus result in an increase in activity. Support for this was found in the 44 K (with an additional lysine introduced to the C-terminus) and T20K mutants of sakacin P, which show increased cell binding and potency relative to the wild-type peptide [52].

Approaches to stabilizing other classes of bacteriocins may have potential for use with class IIa bacteriocins. Due to their composition, proteolytic cleavage of bacteriocins in the gastrointestinal tract represents a major hurdle for any attempts to control gastrointestinal infections. Careful alteration of trypsin recognition sites in class IIb bacteriocin salivaricin P had only minor effects on activity [53]. Chemically synthesized peptides with incorporated d-amino acids may be similarly expected to render the peptide less susceptible to proteolytic cleavage. Analogues of class IIb bacteriocin lactococcin G were synthesized with the N- and C-terminal residues replaced with d-amino acids, which decreased their susceptibility to exopeptidases without much effect on activity [54]. However, the extent of incorporation of d-amino acids has limitations. The enantiomer of leucocin A was synthesized containing exclusively d-amino acids, but it was found to be largely inactive [55]. This may be rationalized based on a chiral interaction between class IIa bacteriocins with the MPT complex [16]. Nonetheless, these methods may be valuable for stabilizing class IIa bacteriocins.

Much work is focused on using biological means to create bacteriocin analogues. Mutagenesis of bacteriocins can be readily achieved, and large quantities of a desired mutant are readily available through recombinant expression. However, the biological production of analogues suffers the restriction of the proteogenic amino acid library. As a contrast, chemical peptide synthesis offers a vast array of possibilities for the introduction of nonproteogenic amino acids. Furthermore, unnatural structural features not found in class IIa bacteriocins such as carbocyclic rings and d-amino acids are feasible. However, chemical peptide synthesis is not trivial, and it is relatively time consuming and costly. For a chemically synthesized bacteriocin to be considered a viable therapeutic agent, it would have to be greatly superior to any biologically producible bacteriocins.

The rational substitution of amino acids in class IIa bacteriocins is one method of creating mutants, and this has provided much information about the structure-function relationship of these bacteriocins. However, for the most part, the mutants have had decreased activity relative to the wild-type bacteriocin. Another common approach uses error-prone PCR to randomly generate mutants in the hope of finding interesting or improved activity. However, approaches such as DNA shuffling [51] of related bacteriocins and NNK scanning [56] have been used to randomly generate vast numbers of mutants, greatly increasing the number of variants produced without requiring a proportionate amount of labour.

NNK scanning allows for the systematic examination of the role of each residue in a peptide. The native codons are replaced one by one with the NNK triplet oligonucleotide, replacing the amino acid coded for by that codon with any of the 20 proteogenic amino acids. This allows for testing a much larger number of variants without requiring the time consuming preparation of each mutant separately. Consequently, the possibility of discovering a mutant with increased potency is greater. NNK scanning has been applied to pediocin PA-1 to examine the importance of each residue for bactericidal activity and was indeed successful in creating some mutants with increased activity [56].

Often, changing one amino acid at a time is not sufficient to create improved variants. It has been suggested that bacteriocins have evolved to be as effective as possible, and so the creation of improved bacteriocins requires greater modification [51]. This is possible using an alternate approach that allows for the swapping of multiamino acid sequences between different class IIa bacteriocins to create a hybrid bacteriocin. This approach has been used to create a DNA-shuffling library in which regions of pediocin PA-1 have been shuffled with 10 other class IIa bacteriocins [51]. Some of the hybrids did indeed show increased activity relative to the wild-type bacteriocins from which they were derived [51]. 

Another approach explored for creating new analogues is to mix the N-terminus of one bacteriocin with the C-terminus of another, thereby creating a chimera. Some chimeras of pediocin PA-1 with other class IIa bacteriocins showed either comparable or greater bactericidal activities to the corresponding natural bacteriocins against certain indicator strains [43, 51]. 

These approaches to randomly generate vast numbers of mutants and hybrids may allow for simplified drug development, facilitating the discovery of novel potent bacteriocins. Furthermore, these approaches enable the development of new bacteriocins tailored towards different strains of pathogenic bacteria.

## 3. Methods for Improving Production of Class IIa Bacteriocins

For any potential therapeutic use of class IIa bacteriocins, an inexpensive method for the production of large quantities must be available. One possibility is to purify class IIa bacteriocins from their natural producer strain, taking advantage of the cationic and hydrophobic characters of these peptides. However, these purifications typically yield only small amounts of purified peptide, often consisting of less than a milligram per liter of culture [49, 57]. However, the outlook is not bleak, as optimizing culture conditions and improving the design of purifications maximizes bacteriocin recovery and permits increased scale. 

Of the class IIa bacteriocins, pediocin PA-1 is most well characterized in terms of optimization of fermentation. Even then, reported yields must be interpreted carefully, as the sensitivity of indicator strains varies and activity tests are performed differently. A variety of different cultivation methods have been used, such as shake-flasks, batch cultures, and fed-batch cultures. Batch cultures in reactors generally allow for greater control over conditions than shake-flask cultures, with precise control of stirring, aeration, and pH. Fed-batch cultures are similar to batch cultures, except a growth-limiting nutrient is added over time, allowing for higher cell densities. 

For the large-scale production of class IIa bacteriocins to be feasible, several conditions must be met. The yield of the fermentation must be satisfactory, otherwise production costs will be high. The growth media must also be inexpensive, although this must be balanced with the bacteriocin yield as the use of more expensive media has been related to improved bacterial growth and bacteriocin production [58].

The highest reported volumetric productivity was accomplished by a repeated-cycle batch culture of *Pediococcus acidilactici* UL5 immobilized in *κ*-carrageenan/locust bean gum gel beads, reaching levels of 133 mg of pediocin PA-1 produced per liter per hour in complex de Man Rogosa and Sharpe (MRS) media [58]. Using less expensive supplemented whey permeate (SWP) media under otherwise identical conditions, 50 mg of pediocin PA-1 was produced per liter per hour. The production of bacteriocins has tended to be much superior in immobilized cell cultures compared to free cell cultures [59], as exemplified by the greater than tenfold increase in production of pediocin PA-1 under immobilized conditions [58]. Naghmouchi et al. have published an informative literature summary of recent work on fermentation yields of pediocin PA-1 [58].

Bacteriocin-producing fermentations have been tested in a large variety of media as an attempt to minimize production costs. Waste from the food industry especially has been investigated as an inexpensive alternative to complex growth media. Examples of this include mussel-processing waste [60, 61], whey permeate [58, 62–64], trout and squid viscera, and swordfish muscle [65]. Complex growth media tend to be composed of a mixture of nutrients tailored to certain types of bacteria to meet their specific nutritional requirements, while industrial effluents are not so optimized [63, 66]. 

Although the production of large amounts of bacteriocin is feasible, the purification of these peptides is another matter. A review by Carolissen-Mackay et al. discusses previous purification approaches for bacteriocins [57]. Many purification protocols provide poor yields of bacteriocin with recoveries of under 20% [57]. These poor yields are likely due to unoptimized protocols requiring a large number of steps. More recently, several general protocols have been published specifically for the purpose of purifying class IIa bacteriocins [67–69]. For the industrial-scale production of bacteriocins required for therapeutic use, an efficient, inexpensive, and scalable purification scheme with high recovery is needed. 

Commonly, the purification of class IIa bacteriocins requires precipitation and centrifugation steps. The latter represents a major bottleneck when attempts are made to increase the scale of production. Furthermore, ammonium sulfate precipitations are frequently a source of loss of material, yielding only 40% ± 20% for a reported pediocin PA-1 purification [67]. Using an initial ion-exchange chromatographic step to concentrate the bacteriocin directly from the culture media is a possible solution [67].

More recent general purification schemes generally follow a similar sequence, taking advantage of the cationic and hydrophobic character of class IIa bacteriocins. First, the culture supernatant is passed through a cation-exchange column [67–69] although loading the whole bacterial culture to avoid centrifugation has been reported [67]. Following this step, the eluate is further purified using hydrophobic interaction chromatography, yielding greater than 90% pure bacteriocin in only two steps [67, 69]. HPLC may also be used to further clean up the sample at this stage [68]. These purifications allow for the acquisition of purified bacteriocin in only a few hours [67], with bacteriocin recovery rates reported ranging from 60% [68] to greater than 80% [67].

The development of antibodies capable of recognizing bacteriocins has allowed for an alternate approach to purification, namely, immunoaffinity chromatography [70–73]. Indeed, this approach has been used to purify divercin V41, piscicocin V1b, enterocin P, and pediocin PA-1 from culture supernatant in a single step. Although reported yields are sparse, the recovery of enterocin P was 44% [71], while 53% of pediocin PA-1 was retained [73]. Although pure bacteriocin is obtained after a single step, superior yields have been reported for lengthier procedures [67], and the immunoaffinity purification requires costly noncommercial antibody-conjugated resins.

Another notable purification approach uses triton X-114 phase partitioning, which has been applied to the purification of divercin V41 [74]. This approach does not require removal of bacterial cells from the culture, thereby enabling collection of the bacteriocin normally lost adhered to the cell pellet. After the two phases partition, the detergent rich phase is removed, diluted, and loaded on to an ion-exchange column. Purified bacteriocin is simply eluted from the column, with a recovery of greater than 55% [74]. 

All of these reported purifications have unique advantages and drawbacks. However, the focus has shifted to the large-scale production of bacteriocins instead of purifying only enough for characterization. These approaches have focused on attaining improved yields in fewer steps with mostly scalable steps.

### 3.1. Heterologous Expression

As an alternative to purification from the natural producer, the recombinant expression of bacteriocins offers a promising means for producing the large amounts of material required for any potential therapeutic use. There have been many reports of the heterologous expression of class IIa bacteriocins in many different hosts, although the focus has been on Gram-positive lactic acid bacteria phylogenetically similar to the producer strain. Gram-negative bacteria such as *Escherichia coli *and yeast expression platforms such as *Saccharomyces cerevisiae* have also been used as expression hosts.

The subject of the heterologous expression of mature bacteriocins in lactic acid bacteria has been summarized in an excellent review by Rodríguez et al. [89], which also discusses heterologous bacteriocin production in *E. coli* and other bacterial strains. Although some of these expression systems allow for the secretion of active bacteriocin into the culture supernatant, the quantity of bacteriocin obtained from these cultures tends to be lower than from the natural producer strain. As such, this is not yet suitable for the large-scale production of bacteriocins required for any potential therapeutic use. However, these heterologous producers may be suitable for food preservation as many lactic acid bacteria are generally recognized as safe. Furthermore, these organisms are capable of simultaneously producing multiple different bacteriocins allowing for a greater spectrum of activity in addition to the possibility of overcoming the development of resistance [90–92]. However, the use of genetically modified organisms in food products is still a contentious issue.

The heterologous expression of bacteriocins as fusion proteins in *E. coli* has been successfully used for the production of larger amounts of bacteriocin than obtained using other approaches. In particular, the commercial strain *E. coli* Origami (DE3) has been used extensively in this area. Mutations in the genes encoding the glutathione and thioredoxin reductases of this strain allow for the facile formation of the conserved disulfide bridge in the host cytosol.

Additionally, the heterologous expression of class IIa bacteriocins in *E. coli* as fusion proteins offers many advantages. A summary of the reported use of fusion proteins partnered with class IIa bacteriocins is presented in Table 1. Fusions with affinity labels, such as hexahistidine tags, allow for simplified purification protocols. Additionally, some fusion partners help solubilize the bacteriocin and prevent the desired peptide from forming inclusion bodies, allowing for increased bacteriocin production. The presence of a fusion partner also decreases the antimicrobial activity, avoiding possible toxic effects on the host cell [93–95], although there are exceptions [96]. Thioredoxin in particular is useful as a fusion partner. Beyond circumventing the formation of inclusion bodies, a thermostable thioredoxin fusion allows for a thermal coagulation purification step [97]. This has been used to remove high molecular weight contaminants during the purification of carnobacteriocins BM1 and B2 [50]. Furthermore, thioredoxin may even assist in the formation of the conserved N-terminal disulfide bridge [97].

Expression of a bacteriocin solely with a hexahistidine tag has been reported for pediocin PA-1 [104]. However, this recombinant pediocin PA-1 was found to be toxic to the *E. coli* producer. The purification was complicated by the requirement for denaturing conditions to allow for immobilized-metal affinity chromatography, although the His-tagged peptide was antimicrobially active. Furthermore, expression of small-sized recombinant peptides in *E. coli* is complicated due to the presence of proteases [105]. The expression of bacteriocins with a larger fusion partner is likely to be advantageous.

The conditions used for fermentations have a significant impact on the amount of fusion protein produced. The final yield of purified bacteriocin is influenced by the purification protocol as well as the method used for fusion protein cleavage. Simple shake-flask cultures have been reported most, although many of the reported yields are admittedly not optimized. Piscicolin 126 was cleaved from a thioredoxin fusion yielding 26 mg per liter [99], while a divercin RV41-thioredoxin fusion yielded between 18 and 23 mg of purified peptide per liter of culture [96, 106]. 

High-cell density *E. coli* cultures have also been explored as a means to further increase the production of bacteriocin fusion proteins [50, 106]. The level of production of a recombinant divercin V41-thioredoxin fusion in batch and fed-batch cultivation has been compared to shake-flask cultures [106]. Compared to the yield of 18 ± 1 mg obtained per liter in shake flask cultures, batch and fed-batch yielded 30 ± 2 and 74 ± 5 mg per liter, respectively. However, the highest yields reported are for carnobacteriocins BM1 and B2. These bacteriocins were expressed as thioredoxin fusions in a fed-batch fermentation induced with lactose. The final yields reported are around 320 mg of carnobacteriocin BM1 and carnobacteriocin B2 per liter of the culture, fourfold greater than previous reports [50].

A disadvantage of using bacteriocin fusion proteins is the necessary cleavage and further purification required to get pure bacteriocin. Enzymatic cleavage methods are the most common approach, while chemical methods have also been used. Enzymatic approaches offer the advantage of more specific recognition sites and are thus more compatible with most bacteriocin sequences—although the enzyme recognition is not always infallible [22]. 

Cyanogen bromide (CNBr) is a common chemical means of cleaving fusion proteins, selectively cleaving on the C-terminal side of methionine residues. However, methionine is found in many class IIa bacteriocins. This has been circumvented with carnobacteriocin BM1, wherein methionine-41 was substituted with a valine residue with some impact on activity [50]. However, CNBr has significant advantages over proteases: cost and cleavage efficiency. Besides being much less expensive, the cleavage efficiency of CNBr has been reported to be up to twofold higher than enterokinase [50, 95].

An alternative approach for fusion protein cleavage requires the presence of the amino acid sequence Asp-Pro just N-terminal to the desired sequence. This cleavage method requires heating under strongly acidic conditions, as has been applied for the cleavage of a divercin V41 thioredoxin fusion [106]. This offers an inexpensive method to remove the fusion tag, although these may seem like unsuitable conditions for a peptide. However, class IIa bacteriocins tend to be stable at elevated temperatures and in acidic conditions [49]. 

The use of the intein-chitin-binding domain as a fusion partner allows for circumvention of several of the issues related to fusion proteins. Following the binding of the fusion protein on a chitin resin, cleavage is induced with DTT, resulting in elution of purified bacteriocin without requiring purification from the fusion partner. This has been successfully applied for a variety of class IIa bacteriocins, although the yields have not been very substantial [101].

Yeast expression platforms are another option for the production of class IIa bacteriocins. *Saccharomyces cerevisiae* has been used as an expression host for pediocin PA-1 [107] and plantaricin 423 [108]. Antimicrobial activity was indeed observed, and colonies of yeast growing on agar inoculated with *Listeria *showed zones of inhibition. However, very little antimicrobial activity was observed in the supernatant [107, 108]. This low level of activity may be attributed to the bacteriocin remaining associated with the fungal cell wall [107].

The use of *Pichia pastoris* as an expression host is more promising, showing much higher levels of activity. The levels of enterocin P produced by *P. pastoris* reached levels up to 28 mg/L, almost four-fold higher than that produced by the natural producer strain, *Enterococcus faecium* P13 [109]. However, the final purified yield of enterocin P from *E. faecium * P13 was still superior, demonstrating that improved purification methods are required to take advantage of any increased production. Class IIa-like bacteriocin hiracin JM79 has also been expressed in *P. pastoris*, with similar issues [91]. Although the quantified amount of bacteriocin exceeds that of the natural producer, the observed antimicrobial activity was found to be relatively smaller. Neutral proteases have been suggested as a possible reason for this discrepancy, and bacteriocin amounts may be overestimated due to the nature of the quantitative techniques used [91, 109]. Furthermore, the activity of pediocin PA-1 produced by *P. pastoris *was found to be inhibited by the presence of a collagen-like material, which appeared to be covalently bound to the pediocin [110].

## 4. *In Vivo* Utilization of Class IIa Bacteriocins

As previously discussed, most published work regarding the *in vivo* use of bacteriocins has focused on the introduction of probiotic bacteria to the gastrointestinal tract, where they will potentially secrete bacteriocins. Considerably less research has been done on the administration of purified bacteriocin. The use of probiotic strains may prove beneficial as a prophylactic, but the use of purified bacteriocins appears to be superior for countering an established infection. This has been demonstrated by the administration of either pediocin PA-1 or *Pediococcus acidilactici *UL5, a producer of pediocin PA-1, to mice infected with *L. monocytogenes* [40].

An important concern regarding the use of antibiotics is the effects they have on the microbiota of the body. The presence of commensal bacteria offers an invaluable barrier to infection by opportunistic pathogens. Ideally, an antimicrobial agent should specifically target the pathogenic bacteria with only minimal impact on the natural flora. In fact, the spectrum of activity for class IIa bacteriocins may be extremely well suited for targeting specific pathogens such as *L. monocytogenes in vivo*. Pediocin PA-1 has been tested *in vitro* against screens of common human intestinal bacteria such as bifidobacteria [75, 76], and at the concentrations tested, no antagonistic activity was observed against any of the assayed organisms. This differs from class I lantibiotics nisin A and nisin Z, both of which inhibited the majority of Gram-positive strains tested [75, 76]. Similarly, culture supernatant containing pediocin PA-1 was found to only inhibit one strain of a screen of common gut bacterial species [77]. Furthermore, an *in vivo* study of pediocin PA-1 in a mouse model showed no effect on the composition of the mouse intestinal flora. Likewise, purified pediocin PA-1 fed to rats did not affect the majority of their microbiota [77]. As a contrast, antibiotics such as penicillin and tetracycline strongly inhibited most of the common intestinal microbiota tested [76].

Two different routes of bacteriocin administration to fight *L. monocytogenes* have been tested in mouse models: intravenous [78, 79] and intragastric [40]. The effects of pediocin PA-1 have also been studied in uninfected mice [80], rabbits [80], and rats [77]. The suitability of the route depends on the nature of the pathogen being targeted, as well as the stage of the infection. However, as peptides, bacteriocins face challenges related to their structure not shared by many antibiotics.

Piscicolin 126, recombinant divercin RV41 (DvnRV41), and structural variants of DvnRV41 were all administered intravenously to mice previously or soon to be infected with *L. monocytogenes* [78, 79]. In the control, the intravenous and intraperitoneal injection of these bacteriocins into healthy mice resulted in no visible ill effects [78, 79]. The efficacy of intravenous administration of bacteriocin was tested both prior to and after the intravenous introduction of *Listeria*. Injection of bacteriocins was effective both 15 minutes prechallenge and 30 minutes postchallenge. However, administration of piscicolin 126 24 hours postchallenge showed no significant reduction in listerial counts. Both of these experiments used only 2 *μ*g of purified bacteriocin. The intracellular nature of *Listeria* as a pathogen may explain the lack of sensitivity observed following bacteriocin administration 24 hours postchallenge [25].

A possible concern with the intravenous administration of peptides is the possibility of an immune response. Foreign peptides are often antigenic, and the introduction of these peptides could trigger an immune response. To test this, pediocin AcH was intraperitoneally introduced into mice and rabbits to determine its antigenic properties. However, it did not elicit an antibody response and appears to be nonimmunogenic [80]. In fact, approaches to develop antibodies to class IIa bacteriocins have required conjugation to polyacrylamide gel [81] or carrier proteins such as keyhole limpet hemocyanin [70, 71, 82, 83].

The intragastric administration of bacteriocins suffers from its own set of problems. Bacteriocins are subjected to harsh environments designed precisely for the proteolytic cleavage of peptides and proteins. Class IIa bacteriocins are susceptible to common digestive proteases. Furthermore, the stomach is a highly acidic environment. However, class IIa bacteriocins tend to be relatively stable to acidic conditions, and pediocin PA-1 was stable at pH 2.5 for at least two hours [84]. 

The stability of bacteriocins in the gastrointestinal tract has been examined by passing purified pediocin PA-1 through an artificial system mimicking the human stomach and small intestine [85]. Pediocin PA-1 retained some activity after 90 minutes in the artificial gastric conditions, while all activity was lost once the sample was in the duodenal compartment. It was suggested that pancreatin in the duodenum was responsible for the ultimate cleavage of the pediocin PA-1, while a combination of pepsin and low pH may be responsible for the decrease in activity observed in the gastric chamber. This is in agreement with *in vivo* results, as pediocin PA-1 fed to rats was not detected in their fecal samples [77]. Despite this, the intragastric administration of pediocin PA-1 has been proven effective for decreasing the load of *L. monocytogenes* in a mouse model [40]. Furthermore, encapsulation may preserve bacteriocin potency in the gastrointestinal tract, although this has not been reported for class IIa bacteriocins as of yet. However, encapsulating the lantibiotic nisin in liposomes has shown some success [86–88].

The intragastric administration of pediocin PA-1 to mice infected with *L. monocytogenes* has been examined [40]. Treatment with 250 *μ*g of pediocin PA-1 a day for three consecutive days resulted in a 2-log reduction in fecal listerial counts. *L. monocytogenes* generally crosses the epithelial barrier once it enters the small intestine and then spreads to the liver, spleen, and central nervous system [25]. This bacteriocin treatment was found to decrease the amount of *L. monocytogenes* reaching the liver and spleen [40]. 

### 4.1. Toxicity

An advantage that bacteriocins hold over some other antimicrobial therapies is their composition. These peptides can be easily broken down to simple nontoxic amino acids that are metabolized, although this also means that they may not be as long-lasting compared to antibiotics. However, information regarding the *in vitro* cytotoxicity of class IIa bacteriocins is relatively limited. The cytotoxicity of pediocin PA-1 was tested against simian virus 40-transfected human colon cells and Vero monkey kidney cells [111]. At the levels tested, pediocin PA-1 did show cytotoxic effects on both cell lines, with a bacteriocin dose of 700 AU/mL (likely around 10–20 mg/mL) causing a decrease of greater than 50% on the viable cell counts. Lower dosages also affected the viable cell count, although this was not as dramatic. However, combinations of carnobacteriocins BM1 and B2 at concentrations 100-fold higher than required for antimicrobial activity displayed no significant cytotoxic effects to the human gastrointestinal Caco-2 cell line [112]. The means of bacteriocin production and purification must also be considered with respect to potential toxic effects. Although this paper focuses on the administration of purified bacteriocin only, there still may be the possibility of toxic contaminants retained in the bacteriocin sample, which could confuse any toxicity results obtained. 

Based on the differing results obtained from these two *in vitro* studies, further work must be done to carefully examine what amounts of class IIa bacteriocins can be used safely without cytotoxic effects. However, it is promising that mouse and rabbit models did not show detrimental effects from bacteriocin introduction [40, 79, 80].

### 4.2. Resistance Mechanisms

As with all therapeutic antibiotics, the development of resistance to class IIa bacteriocins in pathogenic bacteria is a critical issue to consider. This topic has been the subject of a recent review by Kaur et al. [113]. Much evidence has shown that the sensitivity of a bacterial strain to class IIa bacteriocins is dependent on the presence of a mannose phosphotransferase (MPT) transporter system [16, 114–116]. Additionally, there is evidence that nonclass IIa bacteriocin lactococcin A also requires MPT as a receptor [17]. Decreased expression levels of MPT have been implicated in resistance to class IIa bacteriocins in many strains of *L. monocytogenes* insensitive to bacteriocins [114].

Beyond decreased receptor expression, *L. monocytogenes *and other susceptible strains have developed other resistance mechanisms. Multiple mechanisms may be operative at once contributing to an overall resistant phenotype. Modifications of the bacterial membrane have been implicated as another source of bacterial resistance. Alterations of the bacterial membrane, such that the acyl chains of phosphotidylglycerols are shorter and more unsaturated, affect membrane fluidity and the efficiency of bacteriocin insertion [117, 118]. Several other observed cell surface adaptations have been implicated in resistance, such as increasing the net positive charge on the membrane and lysinylation of membrane phospholipids [119]. 

Of special concern is the cross-resistance that has been observed for bacteriocins from different classes. For example, a strain of *L. monocytogenes* has shown resistance to nisin, pediocin PA-1, and leuconocin S, bacteriocins from three separate classes [120]. Based on this, the prospect of using multiple bacteriocins to overcome resistant strains may not be entirely feasible. Like other antibiotics, bacteriocins need to be used judiciously to minimize the spread of resistant phenotypes.

## 5. Conclusions

Class IIa bacteriocins are antagonistic to many important human pathogens. These bacteriocins have the ability to target a relatively narrow range of bacteria without affecting much of the natural microbiota of the body, which is an important advantage, especially when compared to other antibiotics. Although these bacteriocins do not target as many pathogens as other antibiotics, they have the potential to perform a very specific role. Having another tool to combat infections is especially important with consideration of the ever-growing problem of antibiotic resistance.

Although relatively little has been published about the actual *in vivo* use of class IIa bacteriocins to control bacterial infections, what is known is promising. Preliminary experiments have shown these bacteriocins to be effective at fighting *L. monocytogenes* infections in mouse models. 

Now, more is known about the mode of action of bacteriocins, and attempts at engineering bacteriocins with greater potency and stability have been successful. Compared to some other classes of bacteriocins, class IIa bacteriocins are especially suitable for facile recombinant production and the preparation of analogues. Improved fermentation conditions in combination with scalable efficient purifications are now known, allowing for the industrial-scale production of pure bacteriocin. The recombinant production of class IIa bacteriocins as a variety of fusion proteins in *E. coli* has also been successful, allowing for the production of even greater amounts of bacteriocin.

The application of class IIa bacteriocins as therapeutic agents is a rapidly developing area, and there is still much to investigate. In particular, determination of their efficacy against pathogens other than *L. monocytogenes* is open for exploration and would further reveal their potential for therapeutic use. In addition, it would be informative to test these bacteriocins against a wider range of targets beyond Gram-positive bacteria, as they have displayed unexpected activity. 

The methodology is now in place to produce and purify large amounts of class IIa bacteriocins. The preliminary characterization that has been done reveals that this class of bacteriocins possesses several desirable and useful properties as *in vivo* antimicrobial agents. What remains now is to use that knowledge to fully explore the suitability of these peptides as *in vivo* antibiotics.

## Figures and Tables

**Table 1:** Fusion partners used for class IIa bacteriocins.

Fusion partner	Bacteriocin
Thioredoxin	Pediocin PA-1 [95, 98], carnobacteriocins BM1 and B2 [50], divercin V41 [96], enterocin P [72], piscicolin 126 [99]

Maltose-binding protein	Carnobacteriocin B2 [93] and its precursor [22], Pediocin AcH [100]

Intein-chitin-binding domain	Piscicolin 126, divercin V41, enterocin P, pediocin PA-1 [101]

Dihydrofolate reductase	Pediocin PA-1 [94]

Xpress tag	Pediocin PA-1 [102]

Cellulose-binding domain	Enterocin A [103]

Hexahistidine tag	Pediocin PA-1 [104]
